# Energy metabolic reprogramming regulates programmed cell death of renal tubular epithelial cells and might serve as a new therapeutic target for acute kidney injury

**DOI:** 10.3389/fcell.2023.1276217

**Published:** 2023-11-20

**Authors:** Limei Zhao, Yajie Hao, Shuqin Tang, Xiutao Han, Rongshan Li, Xiaoshuang Zhou

**Affiliations:** ^1^ The Fifth Clinical Medical College of Shanxi Medical University, Taiyuan, Shanxi, China; ^2^ The Third Clinical College, Shanxi University of Chinese Medicine, Jinzhong, Shanxi, China; ^3^ Department of Nephrology, Shanxi Provincial People’s Hospital, The Fifth Clinical Medical College of Shanxi Medical University, Taiyuan, Shanxi, China

**Keywords:** acute kidney injury, renal tubular epithelial cells, energy metabolism, programmed cell death, therapeutic

## Abstract

Acute kidney injury (AKI) induces significant energy metabolic reprogramming in renal tubular epithelial cells (TECs), thereby altering lipid, glucose, and amino acid metabolism. The changes in lipid metabolism encompass not only the downregulation of fatty acid oxidation (FAO) but also changes in cell membrane lipids and triglycerides metabolism. Regarding glucose metabolism, AKI leads to increased glycolysis, activation of the pentose phosphate pathway (PPP), inhibition of gluconeogenesis, and upregulation of the polyol pathway. Research indicates that inhibiting glycolysis, promoting the PPP, and blocking the polyol pathway exhibit a protective effect on AKI-affected kidneys. Additionally, changes in amino acid metabolism, including branched-chain amino acids, glutamine, arginine, and tryptophan, play an important role in AKI progression. These metabolic changes are closely related to the programmed cell death of renal TECs, involving autophagy, apoptosis, necroptosis, pyroptosis, and ferroptosis. Notably, abnormal intracellular lipid accumulation can impede autophagic clearance, further exacerbating lipid accumulation and compromising autophagic function, forming a vicious cycle. Recent studies have demonstrated the potential of ameliorating AKI-induced kidney damage through calorie and dietary restriction. Consequently, modifying the energy metabolism of renal TECs and dietary patterns may be an effective strategy for AKI treatment.

## 1 Introduction

Acute kidney injury (AKI) is a pressing global health issue characterized by a swift decline in renal function, leading to elevated mortality and prevalence rates (Liu et al., 2020). AKI can be triggered by various factors such as trauma, sepsis, surgery, or drug toxicity, with ischemia-reperfusion injury (IRI) being the primary cause. IRI disrupts the cellular redox balance and triggers excessive generation of reactive oxygen species (ROS) in the kidneys during reperfusion, leading to a series of events, including mitochondrial damage, energy consumption, apoptosis, and necrosis of renal tubular cells (Han et al., 2017). Furthermore, incomplete recovery from AKI may lead to renal fibrosis, increasing the risk of chronic kidney disease (CKD) and end-stage renal disease (ESRD) (Lee et al., 2022). Presently, no specific treatment exists for the prevention of AKI, enhancing recovery, or improving the long-term prognosis of AKI, including CKD, ESRD, or death (Yang et al., 2006). Therefore, there is an urgent need to identify novel therapeutic targets for AKI treatment.

The kidney is the second largest metabolic organ in the human body with abundant mitochondria, second only to the heart (O'Connor, 2006; Pagliarini et al., 2008). Under normal physiological conditions, the kidney requires a large amount of energy to maintain its functions. Tubular epithelial cells (TECs) are one of the primary sites of AKI injury, and under pathological conditions, their mitochondria suffer severe damage, leading to disruptions in oxidative phosphorylation and energy metabolism disorders (Bhargava and Schnellmann, 2017). Consequently, the utilization of metabolic substrates (such as glucose, amino acids, fatty acids (FAs), ketone bodies, citric acid, and lactate) in TECs undergoes alterations to adapt to the pathological environment during AKI (Bhargava and Schnellmann, 2017). It should be noted that this metabolic reprogramming in TECs during AKI serves as a double-edged sword. Current evidence suggests that this reprogramming may represent a protective mechanism for surviving TECs to adapt to the pathological environment. However, prolonged inhibition of FAO and increased glycolysis can influence kidney outcomes and promote the transition of AKI to CKD through multiple molecular mechanisms (Simon and Hertig, 2015). Additionally, numerous studies have found that changes in energy metabolism can also regulate programmed cell death (Fougeray et al., 2012; Wu et al., 2021). Therefore, this article presents a comprehensive summary of the changes in the energy metabolism of TECs and the interplay between these energy metabolism alterations and programmed cell death, offering insights into the diagnosis and treatment of AKI.

## 2 Energy metabolism of renal tubular epithelial cells in healthy kidneys and acute kidney injury

### 2.1 Lipid metabolism

#### 2.1.1 Lipid metabolism in healthy renal tubular epithelial cells

**FIGURE 1:**
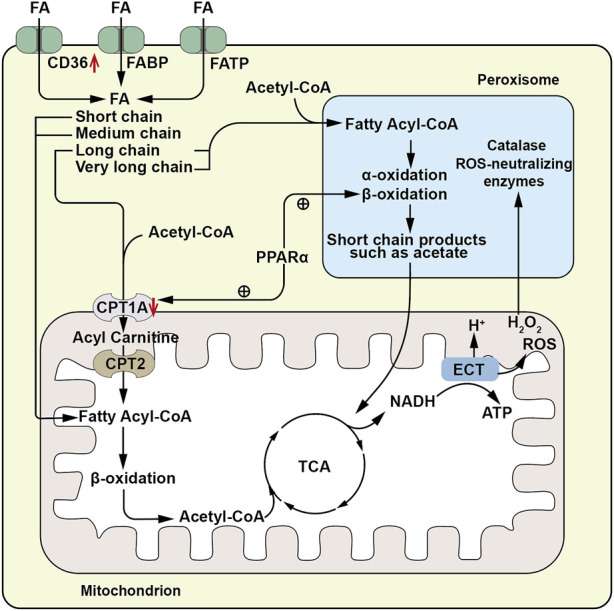
Schematic diagram of fatty acid metabolism pathway. FAs are absorbed by renal tubules via the CD36 receptor, fatty acid binding protein (FABP), and fatty acid transport protein (FATP). Long chain and very long chain FAs are acylated to acyl-CoA by very long chain acyl-CoA synthetase located on the peroxisomal membrane, then enter peroxisomes for beta-oxidation, generating short chain products such as acetic acid. The short chain products enter the mitochondrial TCA cycle for complete oxidation. Short -, medium - and long-chain FAs (FA) are conjugated to CoA by acyl- CoA synthetase on the outer mitochondrial membrane to form fatty acyl-CoA. Long chain acyl-CoA is converted to long chain acylcarnitine by carnitine palmitoyltransferase 1 A(CPT1A) located on the outer mitochondrial membrane, enters the mitochondrial matrix, and is then converted to acyl-CoA by carnitine palmitoyltransferase-2 (CPT2), releasing carnitine. Acyl-CoA undergoes β-oxidation in the mitochondria, producing acetyl-CoA which enters the tricarboxylic acid (TCA) cycle for complete oxidation. The resulting nicotinamide adenine dinucleotide (NADH) and flavin adenine dinucleotide (FADH) enter the electron transport chain (ETC) to provide electrons for generating an electrochemical gradient, leading to ATP production. The ROS and H2O2 generated during this process can enter peroxisomes for metabolism by peroxisomal catalase and other ROS neutralizing enzymes. ↑, Up-regulation of the substance or metabolic pathway in AKI; ↓, Down-regulation of the substance or metabolic pathway in AKI. ⊕, Positive regulatory effect.

The kidney performs a complex reabsorption function, and TECs are the key players responsible for the reabsorption processes involving sodium, water, glucose, and other substances. Similar to the metabolically active cardiomyocyte, TECs relies on fatty acid oxidation (FAO), because per 1 molecule of 16-carbon saturated fatty acids provides 106 ATP units compared to 36 from one molecule of glucose metabolism (Simon and Hertig, 2015). Renal tubules absorb FAs through the cluster of differentiation 36 (CD36) receptor, fatty acid binding protein (FABP), and fatty acid transport protein (FATP) expressed on the plasma membrane (Trimble, 1982; Susztak et al., 2005). Additionally, FAs can be produced through fatty acid synthase in the cytosol or through the metabolism of phospholipids via phospholipase A2 (Simon and Hertig, 2015). Long-chain FAs, such as palmitic acid esters, are the preferred substrates for ATP generation by proximal renal tubular epithelial cells (PTECs). This transportation is facilitated by carnitine palmitoyltransferase 1 (CPT1), located on the outer mitochondrial membrane. CPT1 converts fatty acyl-coenzyme A into long-chain acylcarnitine, allowing it to enter the mitochondrial matrix, where it is converted back to acyl-coenzyme A (acyl-CoA) by CPT2, releasing carnitine. Acyl-CoA undergoes β-oxidation in the mitochondria, and the resulting acetyl-CoA is completely oxidized in the tricarboxylic acid (TCA) cycle. The resulting nicotinamide adenine dinucleotide hydrogen (NADH) and flavin adenine dinucleotide hydrogen (FADH) enter the electron transfer chain (ETC) generating an electrochemical gradient that promotes ATP production (Houten et al., 2016; Gewin, 2021) (Figure 1). Among the three different gene-encoded subtypes of CPT1(a,b and c), CPT1A is most highly expressed in renal PTECs and is crucial for FAO initiation, as it catalyzes the rate-limiting step of the process, making its expression and activity key determinants of proximal renal tubular ATP production (Szeto, 2017).

In the proximal tubules, long-chain FAO occurs not only in the mitochondria but also in the peroxisomes. Peroxisomal FAO operates independently of carnitine and employs very long-chain acyl-CoA synthetase situated on the peroxisomal membrane to catalyze the esterification of very long-chain fatty acids with coenzyme A to generate very long chain acyl-CoAs. However, within peroxisomes, acyl-CoA oxidase (ACOX) remains inactive when presented with substrates containing an acyl group composed of eight or fewer carbon atoms. This restriction impedes the shortening of the carbon chain. Unlike the mitochondria, peroxisomes lack respiratory chain enzymes, and therefore, peroxisomal FAO is not directly coupled with ATP production. However, they metabolize long-chain FAs into acetic acid and other short-chain products with higher hydrophilicity, allowing them to cross the membrane to exit the cell or enter the mitochondria for complete oxidation (Hashimoto, 1996). Additionally, peroxisomes contain abundant catalase and other ROS-scavenging enzymes, protecting PTECS from the toxic accumulation of long-chain FAs and serving as a sink for ROS generated in mitochondria (Vasko, 2016) (Figure 1). Hashimoto (1996) discovered that ACOX dysfunction leads to peroxisomal dysfunction and subsequent accumulation of long chain FAs, confirming the role of peroxisomes. Furthermore, peroxisomal activity varies with different physiological conditions, and peroxisomal proliferation significantly increases the activity of peroxisomal oxidase (Hashimoto, 1996). These findings indicate a cooperative relationship between peroxisomes and mitochondria, supported by observed physical interactions between the two, and the secondary effect of peroxisome function ablation on mitochondrial function (Peeters et al., 2015).

The metabolic pathways of FAO are regulated by reversible post-translational modifications (PTMs) (Rardin et al., 2013), particularly lysine acylation, and the sirtuin deacetylase that removes these PTMs. Among the sirtuins, nuclear/cytosolic sirtuin1 (Sirt1) has been shown to prevent AKI by restoring the number and function of peroxisomes, upregulating catalase, and eliminating renal ROS (Hasegawa et al., 2010). Mitochondrial sirtuin3 (Sirt3) also exerts a renal protective effect by improving mitochondrial dynamics (Morigi et al., 2015). Sirtuin5 (Sirt5) has a unique substrate preference for succinyl-lysine, propionyl-lysine, and glutaryl-lysine among sirtuins. Studies have demonstrated that Sirt5 promotes FAO in mitochondria but inhibits FAO in peroxisomes. Notably, after ischemia- or cisplatin-induced AKI, Sirt5−/− mice showed higher peroxisomal FAO than mitochondrial FAO in PTECs, leading to significantly improved function with reduced tissue damage (Chiba et al., 2019).

#### 2.1.2 Lipid metabolism in renal tubular epithelial cells during acute kidney injury

##### 2.1.2.1 Fatty acid metabolism

Mitochondrial damage in PTECs is a common feature of AKI caused by various etiologies, and individual metabolic pathways are also dysregulated. Studies have shown that FAO is significantly downregulated in all types of AKI, primarily due to the lack of expression of CPT1A, which prevents medium-chain and long-chain FAs from entering the mitochondrial matrix. Overexpression of CPT1A and its activators has been found to alleviate AKI (Idrovo et al., 2012). CPT1A expression is regulated by various factors, including peroxisome proliferator-activated receptor coactivator (PGC)-1 alpha (PGC1α), as well as nuclear receptor family members such as the peroxisome proliferator-activated receptor-alpha (PPARα), PPARγ, estrogen-related receptor alpha (ESRRα), pregnane X receptor (PXR), and farnesoid X receptor (FXR). In AKI, alterations in the expression of these factors affect FAO metabolism. For example, the Kruppel-like factor (KLF15) positively regulates CPT1A transcription and controls FAO metabolism by binding to PPARα, but it is down-regulated in AKI (Piret et al., 2021a). Additionally, ESRRα inhibitors can inhibit the expression of PPARα and CPT1A (Dhillon et al., 2021). PXR-deficient rats exhibit reduced gene expression of CPT1A and medium-chain acyl-CoA dehydrogenase (MCAD) (Yu et al., 2020). Recent studies have also identified a co-regulation between FXR and PPARγ in PTEC FAO. In cisplatin-induced AKI, PPARγ in PTEC is down-regulated, and treating cisplatin-damage PTEC with FXR agonists restores PPARγ expression. PPARγ-specific knockdown mice experience aggravated renal TEC injury and increased lipid droplet formation after cisplatin treatment, with reduced expression of PGC1α, leading to a decrease in CPT1A expression (Fontecha-Barriuso et al., 2020; Fontecha-Barriuso et al., 2022; Xu et al., 2022). PGC1α, a key co-activator factor for peroxisomes, plays crucial roles in peroxisome remodeling and abundance. These discoveries imply a mutual interaction among these factors. Their up-regulation can enhance CPT1A expression, consequently improving mitochondrial FAO and ameliorating acute kidney injury (Hu et al., 2012; Wang et al., 2021). Furthermore, Chiba et al. (2019). upregulated peroxisomal FAO in mice by deleting the lysine deacetylase Sirt5, leading to the preservation and improvement of renal function after cisplatin and IRI. This indicates that upregulating peroxisomal FAO may serve as a novel therapeutic target for AKI. PPAR agonists have been shown to upregulate mRNA expression of peroxisomal FAO enzymes, as well as their metabolic activity and proliferation rate. Therefore, dual improvement of AKI TEC injury may be achieved by upregulating both CPT1A and peroxisomal FAO through PPAR agonists or overexpression of PPAR (Figure 1). However, considering that PPAR regulates many physiological processes in the kidney, its effects on AKI are multifaceted. In addition to their role in protecting the kidneys by regulating energy metabolism, synthetic PPARγ agonists as well as PGC1α agonists may have diverse adverse effects such as fluid retention or blood volume expansion, obesity, heart disease, and liver toxicity (Sauer, 2015; Fontecha-Barriuso et al., 2022). Studies have found that the use of endogenous and natural PPAR agonists can avoid the side effects of excessive activation of PPAR induced by synthetic PPAR agonists (Sharma and Patial, 2022). Therefore, it is crucial to select appropriate PGC1α and PPAR activators and establish the optimal therapeutic time window for PGC1α and PPAR activation.

After IRI, TECs show changes in other genes related to FAs metabolism, in addition to downregulation of the key gene CPT1A associated with FAO. Genes related to FAs transport or storage, such as CD36 and Perilipin 2, exhibit significant upregulation. Similarly, genes involved in FAs synthesis, including fatty acid synthase (FASN), sterol regulatory element-binding transcription factor 1 (SREBF1), and elongation of very long chain fatty acids like protein 1 (ELOVL 1), show a trend of upregulation after IRI. Additionally, genes associated with lipoprotein intakes, such as low-density lipoprotein receptor-related protein 2(LRP2) and very low-density lipoprotein receptor (VLDLR), are increased (Zhang et al., 2018). These results indicate that TECs not only decrease the utilization of FAs but also significantly increase the uptake or storage of FAs during the progression of AKI. This leads to FA accumulation, and evidence shows that the storage of free lipids in lipid droplets can prevent the adverse effects of lipid peroxidation and mitochondrial membrane potential damage caused by the accumulation of free lipids (Zhang et al., 2018; Farmer et al., 2019). The formation of lipid droplets is regulated by the intracellular super transcription factor forkhead box protein O1(FOXO1), which is significantly upregulated in TECs during AKI, along with miR-21-3p, which regulates FOXO1 (Lin et al., 2019). This suggests that the increase in fatty acid synthesis, absorption, and transport in TECs during AKI also contributes to the accumulation of FAs, while the synthesis of lipid droplets is also increasing.

##### 2.1.2.2 Cellular membrane lipid metabolism

The cell membrane lipids encompass various types, including phospholipids, sphingomyelin, glycerolipids, and cholesterol. Among these, phospholipids and sphingomyelin constitute the major components and predominate the cell membrane lipids. Metabolites of phospholipids play an important role in cellular stress response. Lysophosphatidylcholine acyltransferase (LPCAT) catalyzes the conversion of lysophosphatidylcholine (LPC) to phosphatidylcholine (PC) and participates in phospholipid metabolism. LPCAT enzymes include LPCAT1, LPCAT2, LPCAT3, and LPCAT4. Among them, LPCAT3 is widely expressed in various tissues, including the testis, kidney, liver, and small intestine, exhibiting the widest distribution (Yamazaki et al., 2012). In lipopolysaccharide-induced AKI, the expression of miR-124-3p.1 targeting LPCAT3 is decreased, leading to an increase in LPCAT3 activity and, subsequently, an elevation in phosphatidylcholine content in TECs, inducing cellular lipid peroxidation and ferroptosis (Zhang et al., 2022). Another study by Zager et al. (1999) reported an increased percentage of phosphatidylcholine in the renal cortex after 18 h of ischemia, supporting these findings.

**FIGURE 2:**
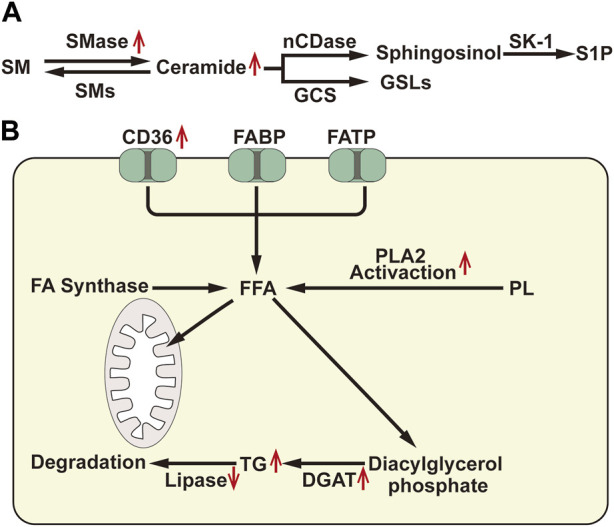
**(A)** Diagram of sphingomyelin metabolism: SM, Sphingomyelin; SMase, Sphingomyelinase; SMs, Sphingomyelin synthase; nCDase, Neutral Ceramidase; GCS, Glucosylceramide Synthase; SK-1, Sphingosine Kinase-1; S1P, Sphingosine-1-Phosphate; GSLs, GlycoSphingomyelin. **(B)** Diagram of fatty acid metabolism: FABP: atty acid binding protein; FATP, fatty acid transport protein; FA, Fatty Acid; FFA, Free Fatty Acid; PLA2, Phospholipase A2; TG, Triglyceride; DGAT, Diacylglycerol Acyltransferase. ↑, Up-regulation of the substance or metabolic pathway in AKI; ↓, Down-regulation of the substance or metabolic pathway in AKI.

Sphingomyelin, as a platform for receptor proteins and lipids, are strategically distributed to specific microdomains of the cell membrane, thereby promoting transmembrane signal transduction. Elevated levels of sphingomyelin have been observed in ischemic kidneys, which may alter the distribution and formation of cytomembrane microdomains in renal TECs, thereby affecting signal pathways and second messenger production. Sphingomyelin can be hydrolyzed by the sphingomyelinase (SMase) family to generate ceramide, a central lipid in the sphingolipid pathway that plays an important role in intracellular processes such as cell proliferation, differentiation, and cell cycle arrest, and is an effective regulator of cell fate and metabolism (Scantlebery et al., 2021). Numerous studies have consistently demonstrated that the upregulation of ceramide synthesis is a common feature in several AKI causes (Laviad et al., 2008; Dahdouh et al., 2014; Valdés et al., 2021; Ma et al., 2022). Inhibiting the accumulation of ceramide by inhibiting SMase can potentially improve renal outcomes across several AKI models (Dupre et al., 2017; Nicholson et al., 2022). Ceramide can be enzymatically converted to sphingosine by neutral ceramidase (nCDase). Subsequently, sphingosine is phosphorylated by sphingosine kinase 1 (SK-1) to generate sphingosine-1-phosphate (S1P) (Figure 2A. A). S1P is a natural sphingolipid ligand of the 5 G protein-coupled receptor (S1P1-S1P5Rs) family, responsible for regulating cell survival and lymphocyte circulation (Bajwa et al., 2015). Several studies propose that S1P1 can stabilize mitochondrial function and alleviate renal TEC injury during IRI (Dupre et al., 2017). Promoting SK-1 synthesis and S1P1 production can prevent ischemia-reperfusion AKI (Park et al., 2012). Moreover, ceramide can also be enzymatically converted to glucosylceramide by glucosylceramide synthase. Inhibitors of glucosylceramide synthase have been shown to increase ceramide levels and decrease glucosylceramide in the renal cortex, rendering mice more susceptible to cisplatin-induced AKI (Nicholson et al., 2022). This indicates that the metabolism of ceramide to glucosylceramide could act as a buffer for renal ceramide and potentially alleviate renal injury. Therefore, reducing neuro amides or inducing S1P generation may represent a promising novel therapeutic strategy for AKI.

Arachidonic acid (AA) is a major component of cell membrane lipids and is mainly metabolized by three active enzymes: cyclooxygenase (COX), lipoxygenase (LOX), and cytochrome P450 (CYP450) enzymes. In the proximal tubules of the kidney, the CYP450 pathway is the primary route for AA metabolism, leading to the formation of 20-hydroxyeicosatetraenoic acid (20-HETE) and 19-hydroxyeicosatetraenoic acid (19-HETE) by ω-19 hydroxylation (Quigley et al., 2000; Wang et al., 2019). Following renal ischemia/reperfusion (I/R), the production of 20-HETE increases, which can activate PPARs and participate in regulating lipid metabolism (Roman et al., 2011; Tanaka et al., 2011). Additionally, 20-HETE has been implicated in prolonging post-reperfusion vasoconstriction and exacerbating IRI in the kidney (Nilakantan et al., 2008). However, in certain instances, 20-HETE can mitigate the effects of IRI by increasing medullary oxygenation (Regner et al., 2009; Roman et al., 2011). Researchers have demonstrated that administration of 20-HETE agonists can prevent the secondary decrease in medullary blood flow and medullary hypoxia after bilateral renal ischemia, thereby reducing the severity of IRI (Regner et al., 2009). On the contrary, some studies have suggested that inhibiting 20-HETE can prevent IRI in acutely uninephrectomized rats (Hoff et al., 2011). Hence, the effect of 20-HETE on AKI may be closely related to the severity of the renal injury.

##### 2.1.2.3 Metabolism of cholesterol and triglycerides

The accumulation of cholesterol in the renal cortex is a delayed consequence observed in various forms of renal damage. After ischemic, toxic, obstructive, or immune-related renal injuries for 18–24 h, the levels of free cholesterol, cholesterol esters, and triglycerides in the proximal renal tubules significantly increase (Zager et al., 1999). Triglyceride level regulation mainly involves the synthesis of free fatty acids (FFAs), diacylglycerol acyltransferase (DGAT)-mediated triglyceride assembly, and the catabolism of triglycerides (Figure 2B). Glycerol and ischemia/reperfusion-induced renal injury can lead to a 20%–35% decrease in lipase activity in the renal cortex, resulting in the accumulation of triglycerides in HK-2 cells. Moreover, HK2 cell injury induced by hypoxia/reoxygenation and lipopolysaccharide (LPS) can activate phospholipase A2 (PLA2), increase the expression of DGAT, and cause mobilization of FAs from the phospholipid pool, consequently increasing triglyceride formation (Johnson et al., 2005). Furthermore, after AKI, cells increase their uptake of FAs, while the consumption of FAs by mitochondria decreases, leading to an increase in substrate for triglycerides and ultimately resulting in triglycerides accumulation.

### 2.2 Glucose metabolism

#### 2.2.1 Glucose metabolism in healthy renal tubular epithelial cells

The kidneys play an important role in the production and metabolism of glucose, with approximately 25% of all glucose released into circulation being produced by the human kidneys (Stumvoll et al., 1995). Glucose has two main sources: one is produced through glycogen breakdown, and the other is through gluconeogenesis. Given the limited glycogen reserves in the kidneys, it is probable that gluconeogenesis serves as the primary mechanism for glucose production. Both insulin signaling and glucose reabsorption by proximal tubular cells play a role in regulating gluconeogenesis. In HK-2 cells, these processes act to inhibit the expression of gluconeogenic genes by deactivating FOXO-1 and PGC1α, respectively (Akhtar et al., 2021). In gluconeogenesis, substrates such as lactate, glycerol, alanine, and glutamine can ultimately produce glucose through the process of glucose-6-phosphate. The primary precursor of renal gluconeogenesis is lactate, which is freely filtered by the glomerulus and subsequently reabsorbed by the renal TECs through the monocarboxylate transporters (MCTs) (Becker et al., 2010). Lactate is then converted to pyruvate by lactate dehydrogenase (LDH). Pyruvate enters the mitochondria and is sequentially converted to oxaloacetate and citrate, which is then exported from the mitochondria as citrate. Finally, citrate is converted back to oxaloacetate by phosphoenolpyruvate carboxykinase (PEPCK), and, ultimately, to glucose. However, although lactate is the predominant substrate for TECs gluconeogenesis, glutamine also makes a significant contribution.

Under normal physiological conditions, renal gluconeogenesis occurs mainly in the proximal tubules, expressing key enzymes required for this process (glucose-6-phosphatase, PEPCK, and fructose-1,6-bisphosphatase). On the other hand, glycolysis is predominant in the distal tubules, which express high levels of glycolytic enzymes (hexokinase (HK), phosphofructokinase (PFK), and pyruvate kinase (PK)) (Legouis et al., 2022). Glucose can be metabolized to pyruvate via glycolysis, and then further oxidized in the TCA cycle or metabolized to lactate. Studies have found that even under aerobic conditions, the distal tubules can also metabolize glucose to lactate (Bagnasco et al., 1985), and it has been discovered that glucose oxidation and ATP generation in the proximal tubules are significantly lower than in the distal tubule segments in rats (Klein et al., 1981). This indicates that glucose is produced by renal gluconeogenesis in the proximal tubule and metabolized by renal glycolysis in the distal renal unit segments.

#### 2.2.2 Glucose metabolism in renal tubular epithelial cells in acute kidney injury

##### 2.2.2.1 Glycolysis and the pentose phosphate pathway

**FIGURE 3:**
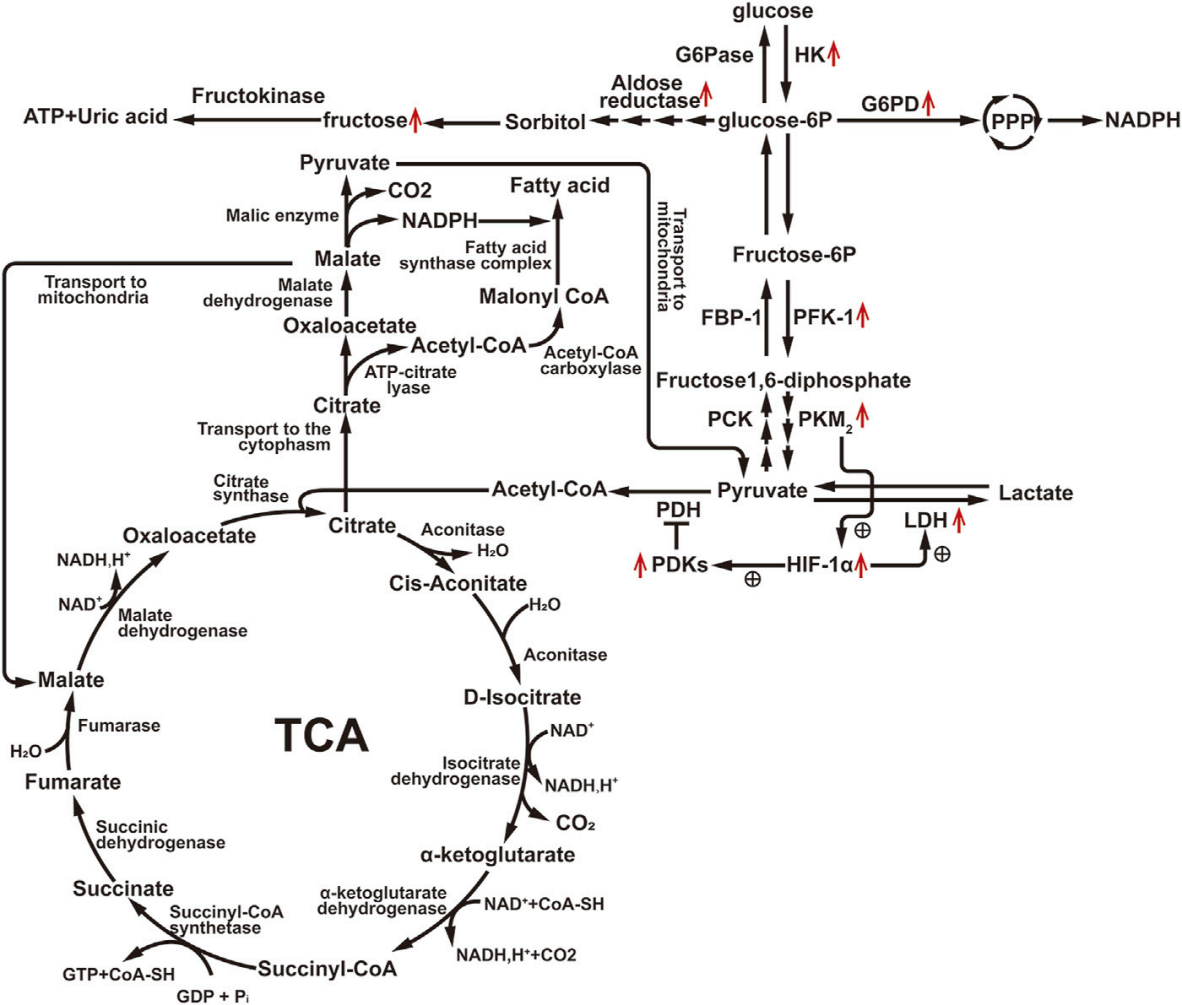
Diagram of glucose metabolism pathway. Illustration: G6Pase, Glucose-6-phosphatase; HK, Hexokinase; G6PD, Glucose-6-phosphate dehydrogenase; FBP-1, Fructose-1, 6-bisphosphatase; PFK-1, Phosphofructokinase-1; PCK, Phosphoenolpyruvate Carboxykinase; PKM2, Pyruvate Kinase M2; PDH, Pyruvate Dehydrogenase Complex; PDKs, Pyruvate Dehydrogenase Kinases; LDH, Lactate Dehydrogenase; HIF-1α, Hypoxia-Inducible Factor-1 alpha. ↑, Up-regulation of the substance or metabolic pathway in AKI; ↓, Down-regulation of the substance or metabolic pathway in AKI. ⊕, Positive regulatory effect.

Recent investigations have elucidated the consequences of impaired FAO in PTECs, resulting in compromised energy production, accumulation of lipids, cellular injury, and eventual fibrosis (Emma et al., 2016). Although under normal physiological conditions, PTEC does not primarily use glucose as fuel, the surviving PTECs during AKI can adapt to FAO defects by increasing glycolysis. Several studies have shown the upregulation of glycolytic enzymes (such as HK, PFK, and PK) at both mRNA and protein levels in sepsis, IRI, and aristolochic acid I (AAI)-induced AKI (Lan et al., 2016; Li et al., 2020). In addition, glucose-6-phosphate, the first metabolite of glycolysis, is produced from glucose under that catalysis of hexokinase, and can also enter the PPP pathway through a series of enzymatic reactions to produce NADPH and 5-phosphoribose (Figure 3), which can be used as substrates for the synthesis of purine and pyrimidine nucleic acids. In AKI, the mRNA expression of several genes encoding PPP enzyme is upregulated (Scantlebery et al., 2021), and the PPP pathway is activated, which in turn provides reducing capacity to antioxidant enzymes (such as glutathione (GSH) reductase) by increasing NADPH level to maintain REDOX homeostasis and reduce oxidative damage (Kim et al., 2009). Elsewhere, Smith et al. (2014) found that LPS-induced HK activation was associated with increased glucose-6-phosphate dehydrogenase activity in the pentose phosphate pathway. The above evidence suggests that both glycolysis and pentose phosphate pathways are upregulated in AKI.

Glycolysis generates only two ATP molecules in the cytoplasm. To produce more ATP, pyruvate, the final product of glycolysis, must enter the mitochondria and be transformed into acetyl-CoA by pyruvate dehydrogenase (PDH), which then enters the tricarboxylic acid cycle to form citric acid. PDH activity can be inhibited by phosphorylation at sites S232, S293, and S300 by pyruvate dehydrogenase kinases (PDKs). Additionally, pyruvate can be converted to lactate by LDH. Hypoxia/reoxygenation-induced elevation of hypoxia-inducible factor-1α (HIF-1α) can upregulate both PDKs and LDH (Eleftheriadis et al., 2022), leading to increased lactate production. In LPS-induced AKI, the expression of pyruvate kinase muscle isoform 2 (PKM2), a critical enzyme responsible for catalyzing the last step of glycolysis, is significantly increased, and PKM2 can directly stimulate HIF-1α transactivation (Luo et al., 2011), indicating that PKM2 can regulate PDKs and LDH to promote lactate generation by activating HIF-1α. Elevated lactate levels and decreased pyruvate levels were also observed in IRI mouse kidney tissues (Wei et al., 2007) (Figure 3). Inhibiting PKM2 to prevent IRI-induced kidney injury may be related to the increase in the pentose phosphate pathway (PPP) flux due to PKM2 inhibition, resulting in increased nicotinamide adenine dinucleotide phosphate (NADPH) generation, thus enhancing GSH and antioxidant enzyme levels (Zhou et al., 2019). This was confirmed by Kim et al. (2015), who demonstrated that activation of p53 during AKI could target TP53-induced glycolysis and apoptosis regulator (TIGAR). They also found that transient activation of TIGAR could suppress PFK-1 activity and the activity of glucose-6-phosphate dehydrogenase (G6PD). In cases of mild ischemic injury, G6PD can quickly recover, and TIGAR can reduce the sensitivity of TECs to injury by inhibiting glycolysis and redirecting glycolysis intermediates to the oxidative branch of the PPP to produce NADPH. However, in severe IRI, TIGAR only inhibits glycolysis and, since PPP and NADPH are not increased due to severe suppression of G6PD, cannot overcome the loss of renal function and histological damage. As a result, under mild ischemic conditions, inhibiting TIGAR may protect mouse TECs by promoting the PPP pathway. Therefore, simply inhibiting glycolysis may not be sufficient to improve AKI, it may require a combination of inhibiting glycolysis and promoting glycolysis intermediates toward the PPP pathway metabolism.

##### 2.2.2.2 Gluconeogenesis

In conditions of fasting and stress, the kidney plays an important role in glucose production, accounting for 40% of the body’s glucose through lactate gluconeogenesis in PTECs (Gerich et al., 2001). During the acute ischemia phase of AKI, gluconeogenic enzyme levels (such as fructose-1,6-bisphosphatase (FBP1), phosphoenolpyruvate carboxykinase (PCK1), PCK2) are decreased, while mRNA levels of glycolytic enzymes (such as HK1, PKM, PFK) are relatively increased, leading to impaired glucose production and lactate clearance (Legouis et al., 2020) (Figure 3). In the early reperfusion phase of AKI, lactate levels are generally lower, possibly due to rapid lactate efflux, as the levels of PDH are decreased, limiting the conversion of pyruvate to acetyl-CoA and promoting its conversion to lactate. However, during the late reperfusion phase of AKI, the depletion of pyruvate is associated with an increase in gluconeogenesis. This observation is further confirmed by experiments where exogenous administration of pyruvate increases the renal cortex glucose content in AKI but not in normal kidneys (Zager et al., 2014). Thus, gluconeogenesis undergoes dynamic changes during the process of ischemia-reperfusion-induced AKI. Changes in gluconeogenesis are regulated by insulin. Studies have found that there is impaired insulin response and insulin resistance in the occurrence of acute kidney injury (Clark and Mitch, 1983; Portilla et al., 2006). The mechanism may be that the up-regulation of FOXO1 expression in renal TECs leads to the block of insulin signaling. Glucose-insulin infusion can reduce renal injury induced by severe I/R in mouse experimental models (Melo et al., 2010), and clinical studies have shown that insulin sensitizing agents can improve AKI (Hu et al., 2012), and intensive insulin therapy for tight control of blood glucose can reduce the incidence of AKI in diabetic patients and critically ill patients (Schetz et al., 2008). This further suggests that AKI is accompanied by an impaired insulin response. Improving insulin response can regulate gluconeogenesis and improve AKI renal function.

##### 2.2.2.3 Polyol metabolism

Glucose metabolism can also generate some polyols, such as sorbitol and xylitol, through a pathway known as the polyol pathway. Under the influence of aldose reductase, glucose is reduced to sorbitol with the supply of NADPH, which can then be further converted to fructose (Figure 3). Normally, aldose reductase expression is rarely detected or absent in the PTECs (Lanaspa et al., 2014), but after AKI, its expression is significantly upregulated within 24 h, leading to the production of fructose in a time-dependent manner (Andres-Hernando et al., 2017). Elevated urine fructose levels have also been observed in AKI patients (Andres-Hernando et al., 2017). Fructose can be metabolized by fructokinase, leading to ATP consumption and uric acid generation (Johnson et al., 2010). Inhibiting fructokinase has been shown to increase ATP levels in the kidneys of AKI mice, reduce local uric acid generation and oxidative stress, and exert a protective effect against AKI. Notably, aldose reductase-deficient mice are unable to produce sorbitol and consequently, exhibit impaired urine concentration mechanisms, leading to polyuria (Andres-Hernando et al., 2017). Thus, targeting fructokinase, either alone or in combination with therapies that reduce renal uric acid production, could represent important treatment strategies for preventing kidney disease or expediting kidney recovery.

### 2.3 Metabolism of amino acids

#### 2.3.1 Metabolism of amino acids in healthy renal tubular epithelial cells

The human kidney plays a major role in maintaining the body’s amino acid pools’ homeostasis. Approximately 50–70 g of amino acids are filtered daily, and the proximal tubules reabsorb almost all of them (97%–98%) (Garibotto et al., 2010). These reabsorbed amino acids can serve as substrates for gluconeogenesis and, after undergoing metabolism, enter the TCA cycle for oxidation. Branched-chain amino acids (BCAAs), including leucine, valine, and isoleucine, are important energy sources for the kidneys. They are metabolized by branched-chain aminotransferase (BCAT) to generate branched-chain α-keto acids, which are then oxidatively decarboxylated by the branched-chain α-keto acid dehydrogenase (BCKDH) complex, producing acyl-CoA. The resulting acetyl-CoA enters the TCA cycle for oxidation (Suryawan et al., 1998). Glutamine metabolism is also important for maintaining acid-base balance, in addition to energy production. Glutamine is metabolized by the proximal tubules to form glutamate, which can be converted to α-ketoglutarate (αKG), an intermediate in the TCA cycle. Ammonium ions resulting from this process are mainly excreted in the urine, serving as a disposable cation to promote acid excretion. The resulting αKG is transported into the mitochondria and converted to succinyl-CoA by the α-ketoglutarate dehydrogenase complex. This succinyl-CoA is further transformed into succinate through the action of succinyl-CoA synthetase. Succinate is then dehydrogenated into fumaric acid, which undergoes hydration to yield malic acid. Malic acid is subsequently transported out of the mitochondria into the cytoplasm, where it is further dehydrogenated to form oxaloacetate. This oxaloacetate is metabolized by phosphoenolpyruvate carboxylase to generate phosphoenolpyruvate (Reshef et al., 2022), a compound that is subsequently converted into glucose or carbon dioxide. This metabolic process generates bicarbonate ions, which are selectively transported into the venous bloodstream, thus partially compensating for metabolic acidosis (Scholz et al., 2021). Moreover, the kidneys play a major role in arginine synthesis and metabolism. Arginine can be metabolized through various pathways, such as generating citrulline and NO via nitric oxide synthase (NOS) in the cytoplasm. It can also produce creatine via mitochondrial arginine-glycine amidinotransferase, guanidinobutyrate via mitochondrial arginase; and urea and ornithine via both cytoplasmic and mitochondrial arginases (Bellinghieri et al., 2006). Ornithine, in turn, serves as a precursor for polyamine synthesis through ornithine decarboxylase. Additionally, tryptophan undergoes extensive metabolism via the kynurenine (KYN) pathway in the proximal tubules, ultimately leading to the *de novo* synthesis of nicotinamide adenine dinucleotide (NAD+). NAD+ is an important cofactor for many enzymes involved in FAO and the TCA cycle, making it essential for overall renal TEC metabolism. Furthermore, phenylalanine, glycine, tyrosine, and serine are also metabolized in the kidney (van de Poll et al., 2004). Taken together, while glucose and FAs are important energy sources for a healthy kidney, amino acid metabolism is undeniably indispensable for maintaining organismal homeostasis.

#### 2.3.2 Amino acid metabolism in renal tubular epithelial cells in acute kidney injury

##### 2.3.2.1 Glutamine

**FIGURE 4:**
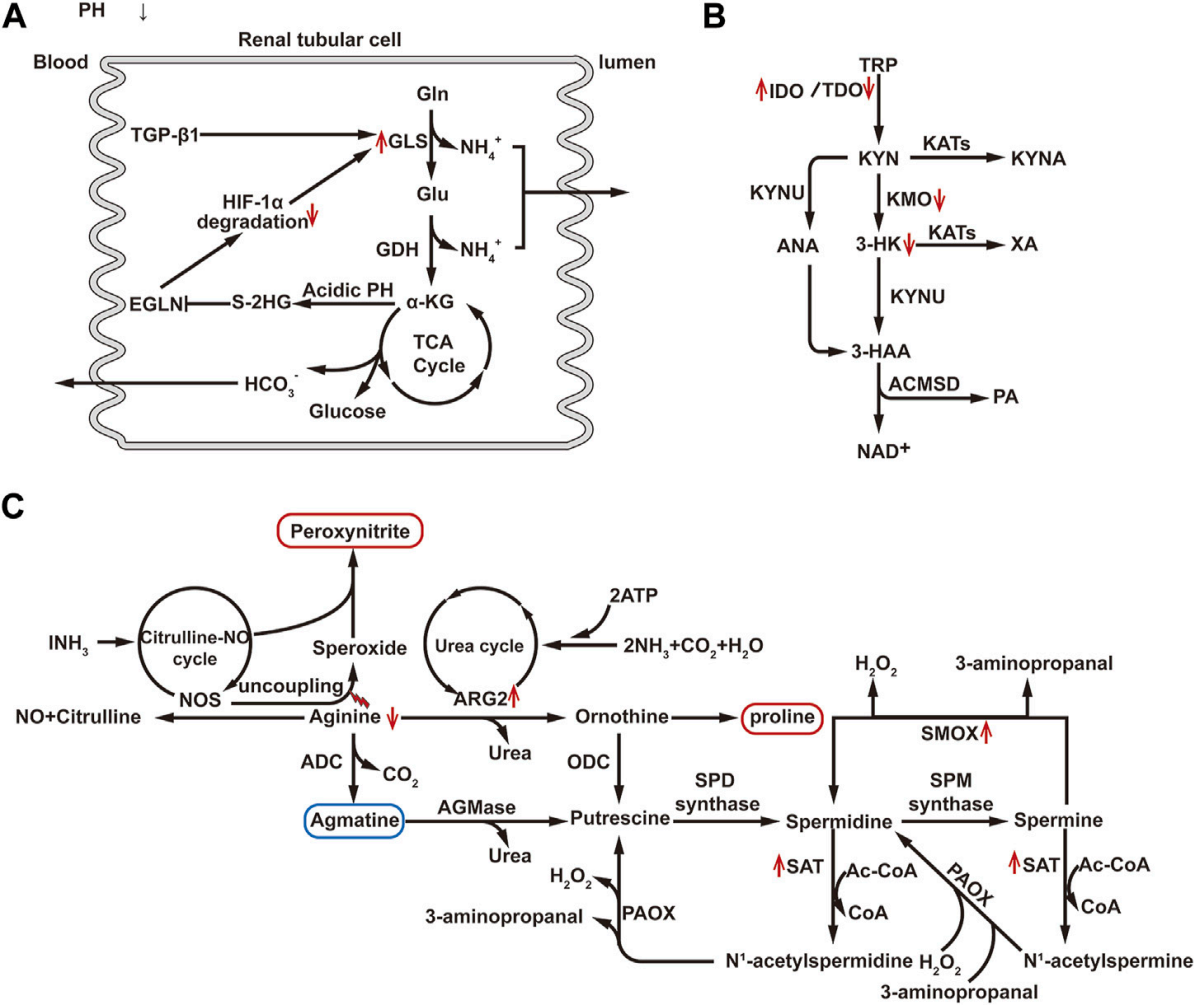
**(A)** Diagram of glutamine metabolism pathway. Gln, Glutamine; Glu, Glutamate; GLS, Glutaminase; GDH, Glutamate Dehydrogenase; HIF-1α, Hypoxia-Inducible Factor-1 alpha; TGF-β1, Transforming Growth Factor beta 1; αKG, Alpha Ketoglutarate; S-2-HG, S-2-hydroxyglutaric acid; EGLN, egg-laying defective nine homolog. **(B)** Diagram of tryptophan metabolism pathway: TRP, Tryptophan; IDO, Indoleamine 2, 3-dioxygenase; TDO, Tryptophan 2, 3-dioxygenase; KYN, Kynurenine; KYUN, Kynurenic Acid; KYNA, Kynurenine Acid; KAT, Kynurenine Aminotransferase; KMO, Kynurenine 3-Monooxygenase; XA, Xanthurenic Acid; 3-HK, 3-Hydroxykynurenine; ANA, Anthranilic Acid; ACMSD, 2-Amino-3-carboxymuconate semialdehyde decarboxylase; PA, Picolinic Acid. **(C)** Diagram of arginine and polyamine metabolism pathway: NOS, Nitric Oxide Synthase; ADC, Arginine Decarboxylase; ARG2, Arginase 2; ODC, Ornithine Decarboxylase; PAOX, Polyamine Oxidase; SSAT, Spermidine/Spermine N^1^-acetyltransferase; SPD synthase, Spermidine Synthase; SPM synthase, Spermine Synthase; SMOX, Spermine oxidase; AGMase, Agmatinase; AC-CoA, Acetyl-CoA. ↑, Up-regulation of the substance or metabolic pathway in AKI; ↓, Down-regulation of the substance or metabolic pathway in AKI.

The kidney normally takes up only a small amount of glutamine. However, during the metabolic acidosis phase of AKI, about one-third of plasma glutamine is taken up and metabolized by the kidney. The increased utilization of glutamine occurs primarily in the proximal convoluted tubules, where a significant increase in glutaminase activity has been observed (Anderson et al., 1982). The mechanism underlying this phenomenon may be related to z-crystal protein, which, under acidic conditions, enhances the binding with glutaminase mRNA sequence, inhibiting ribonuclease recruitment, and stabilizing glutaminase mRNA, resulting in increased glutaminase expression (Curthoys, 2001). Elevated glutaminase promotes glutamine metabolism thereby increase the production of metabolites, such as ammonium and bicarbonate ions, which help compensate for metabolic acidosis during AKI. Additionally, αKG generated from glutamine can supplement the TCA cycle. It has been observed that in IRI, the enhanced expression of transforming growth factor (TGF)-β1 in renal TECs leads to increased expression of glutaminase 1(GLS1), which promotes glutamine decomposition and αKG production (Spurgeon et al., 2005). However, under hypoxia conditions, an acidic PH will promotes the conversion of αKG to 2-hydroxyglutaric acid (2-HG), in which S-2HG constitutes the majority of the 2HG pool and has the effect of inhibiting the egg-laying defective nine homolog (EGLN). Under normoxic conditions, prolyl-4-hydroxylase of EGLN family can label HIF-1α subunits for degradation in an oxygen-dependent manner (Burr et al., 2016). Under hypoxic conditions, the lack of oxygen and the inhibitory effect of S-2HG on EGLN causes HIF-1α accumulation and sustained activation (Cienfuegos-Pecina et al., 2020; Hewitson and Smith, 2021). Activation of HIF-1α, in turn, can regulate the transcription of GLS1 (Stegen et al., 2016). Thus, there appears to be a potential vicious cycle, where the upregulation of HIF-1α leads to an increase in GLS1 expression, resulting in glutamine breakdown and αKG production. and the metabolite s-2-HG of αKG can inhibit prolyl hydroxylase and thus lead to the accumulation of HIF-1α (Figure 4A). HIF-1α, as a key regulatory factor of glycolysis, can induce the shift in cellular energy metabolism towards glycolysis by controlling the expression of several key enzymes involved in glycolysis. As the end product of glycolysis, most of the pyruvate is metabolized into lactate and does not enter the TCA cycle. Therefore, the αKG produced from glutamine metabolism may be essential for supplementing the TCA cycle. Studies in pulmonary fibroblasts using carbon tracing have demonstrated this flux change (Hewitson and Smith, 2021). Bernard et al. (2018) experimentally demonstrated that the primary function of glutamine metabolism in fibroblasts is to supply biosynthetic pathways rather than ATP generation. Elsewhere, Lu et al. (2021) through single-cell sequencing, found that renal TECs undergo phenotype conversion during the repair and proliferation process. Newly generated PTEC circular cells can differentiate into PTEC-S1-new cells, and some of them may subsequently transform into PTEC damage and fibroblasts. In this process, energy metabolism plays an important role. Therefore, it is hypothesized that changes in glutamine metabolism pathways may affect this process, suggesting that intervening in glutamine metabolism could potentially improve kidney injury by regulating the phenotype conversion of newly generated renal TECs.

##### 2.3.2.2 Tryptophan

Tryptophan is an essential amino acid in humans, with 95% of it being metabolized through the kynurenine pathway (KP). The key enzymes in KP include indoleamine 2,3-dioxygenase (IDO) and tryptophan 2,3-dioxygenase (TDO). Both IDO and TDO can be activated by inflammatory factors to degrade tryptophan into KYN. Under normal physiological conditions, tryptophan is mainly metabolized by TDO. However, during inflammation or stress, liver TDO activity is inhibited, and extrahepatic IDO is rapidly activated, leading to a shift in tryptophan metabolism from TDO-dominated to extrahepatic IDO-dominated pathways (Zheng et al., 2019). Subsequently, KYN undergoes further metabolism through three pathways: the NAD branch, converting KYN into 3-hydroxykynurenine (3-HK) and anthranilic acid (ANA), 3-hydroxy anthranilic acid (3-HAA), quinolinic acid (QUIN), and ultimately to NAD, the preferred end product of KP; the picolinic acid (PA) branch, producing PA as the second pathway of KYN metabolism in the kidney and liver. This branch also generates acetyl-CoA, which is further metabolized in the tricarboxylic acid cycle, providing CO_2_ and ATP. The enzyme aminocarboxymuconate semialdehyde decarboxylase (ACMSD) is responsible for shifting KP metabolism from NAD + synthesis to PA production. The third branch of the KYN pathway is the kynurenic acid (KYNA) branch, which produces KYNA and xanthurenic acid (XA) through the mediation of kynurenine aminotransferases (KATs). KYNA exhibits protective effects against inflammation, clears ROS, and demonstrates antioxidant activity. This branch is a minor pathway under physiological conditions, and kynurenine hydroxylase (KYNU) also plays an important role in this pathway (Wee et al., 2021) (Figure 4B). The key enzyme kynurenine-3-monooxygenase (KMO), responsible for converting KYN to 3-HK in human kidneys, is located on the outer membrane of PTECs (Zheng et al., 2019). After AKI, the activity of the IDO enzyme increases in renal TECs leading to an increase in KP decomposition (Krupa et al., 2022). Studies have shown that the expression of KMO in renal TECs decreases in a dose-dependent manner after treatment with cisplatin, resulting in an increase in KYN entering the KYNA and NAD branch (Tan et al., 2021). A longitudinal study has demonstrated that the decrease in 3-HK and the increase in KYNA in AKI patients further support this point (Wee et al., 2021). Furthermore, research has indicated that administering KYNA externally or inhibiting KMO to elevate KYNA levels can alleviate AKI (Arora et al., 2014; Zheng et al., 2019). Inhibition of ACMSD to increase NAD production also showed a protective effect (Katsyuba et al., 2018). In addition, overexpression of PGC1α in the kidney, an important transcriptional co-regulation factor in tryptophan metabolism via KP, can restore mRNA expression of various KP enzymes in AKI mice, increase NAD + levels after IRI, and protect against kidney injury (Tran et al., 2016). These findings suggest that targeting the tryptophan KP pathway may improve AKI.

##### 2.3.2.3 Arginine

The kidney is the primary site of arginine synthesis, responsible for maintaining arginine’s steady state throughout the body. Arginine plays a key role in the urea cycle, acting as a precursor for urea, creatine, and nitric oxide. Under the influence of inflammatory cytokines, arginine metabolism is upregulated, leading to the synthesis of urea, creatine, and proline. In the urea cycle, arginase hydrolyzes arginine into urea and ornithine, facilitating the elimination of toxic ammonium ions (Bellinghieri et al., 2006). Mammals express two subtypes of arginase enzymes: arginase 1 (ARG1, cytosolic) and arginase 2 (ARG2, mitochondrial) (Oates et al., 2019). ARG1 is predominantly expressed in the liver, while ARG2 is primarily expressed in the cortical tubules of the kidney. ARG2 competes with NOS for the same substrate arginine, leading to reduced availability of arginine for NOS. This results in NOS uncoupling, characterized by the generation of superoxide rather than NO, inducing the formation of peroxynitrite. Peroxynitrite acts on proteins or DNA, ultimately leading to cell death or apoptosis. 3-nitrotyrosine (3-NT) is one of the major proteins nitrated by peroxynitrite and is widely used as a marker of nitrosative stress. Studies have shown that from hypoxia to reoxygenation, ARG2 expression is upregulated, and the accumulation of 3-NT gradually increases in HK-2 cells. Inhibition of ARG2 or supplementation with arginine can reduce nitrosative stress after hypoxia/reoxygenation injury to renal TECs, improve renal histological damage, and reduce renal tubular cell apoptosis (Hara et al., 2020; Tanuseputero et al., 2020). Furthermore, the upregulation of ARG2 can also lead to an increase in ornithine levels, and the production of proline from ornithine can promote pathological fibrosis (Hara et al., 2020). These results indicate that the upregulation of ARG2 mediates AKI induced by I/R, and targeting ARG2 may serve as a new therapeutic target for AKI. Furthermore, arginine can also generate guanidinoacetate via mitochondrial arginine decarboxylase, and numerous studies have shown that guanidinoacetate has a protective effect on AKI. Therefore, adjusting arginine metabolism pathways may be a promising strategy for improving AKI. Additionally, Jouret et al. (2016) have demonstrated through metabolomic analysis that arginine metabolism is crucial in the kidney following RI for 48 h.

##### 2.3.2.4 Polyamine

Polyamine biosynthesis relies on the conversion of arginine to ornithine. Ornithine decarboxylase catalyzes the decarboxylation of ornithine, resulting in the production of putrescine. Subsequently, spermidine synthase and spermine synthase add the aminopropyl group to putrescine and spermidine, respectively, ultimately forming spermidine and spermine (Casero and Pegg, 2009). The process involves the acetylation of the N1 position of spermine or spermidine by spermine/spermidine N1-acetylation and N1-acetylpolyamine oxidase (SAT1). Following acetylation, these compounds can be either transported outside the cell or undergo oxidative reversion mediated by polyamine oxidase (PAOX). Additionally, spermine can be directly transformed into spermidine through the action of spermine oxidase (SMOX). During these processes, hydrogen peroxide (H_2_O_2_) and reactive aldehydes are generated (Figure 4C). H_2_O_2_ can cause DNA damage by generating hydroxyl radicals, while reactive aldehydes can damage the integrity of lysosomal and mitochondrial membranes, leading to cell injury through the activation of cell death and apoptosis pathways (Wood et al., 2006). In AKI, activation of p53 enhances the expression and activity of key enzymes involved in polyamine degradation, such as SAT1 and SMOX, thereby enhancing polyamine degradation. Inhibition or ablation of SMOX and SAT1, as well as neutralization of metabolic products (such as H_2_O_2_ and reactive aldehydes), can reduce oxidative stress, inflammation/innate immune responses, and endoplasmic reticulum stress/unfolded protein response, thereby ameliorating renal tubular injury induced by endotoxins, I/R, and cisplatin-induced AKI (Zahedi et al., 2010; Zahedi et al., 2017). However, long-term use of polyamine catabolism inhibitors or ablation of the SAT1 gene has been reported to cause lung injury and induce changes in lipid metabolism (Jain et al., 2018; Yuan et al., 2018). Therefore, additional research is required to confirm the specific effects of improving AKI by inhibiting polyamine metabolism.

##### 2.3.2.5 Branch chain amino acid

Recent studies have revealed a reduction in the activity of BCAA decomposition metabolism enzymes in mice treated with AAI and in cases of septic AKI, leading to decreased decomposition metabolism (Piret et al., 2021b; Standage et al., 2021). This downregulation may hold significant implications for ATP production in the AKI environment when FAO has been impaired, as BCAA decomposition metabolism may promote the TCA cycle through acetyl-CoA and succinyl-CoA. Piret et al. (2021a). discovered that increased expression of Klf6 in AKI injury inhibits the expression of BCAA decomposition metabolism enzymes in HK-2 cells, resulting in decreased mitochondrial ATP production. However, further research is required to confirm whether improving AKI can be achieved by upregulating branch-chain amino acid metabolism.

## 3 Relationship between energy metabolism and programmed cell death in renal tubular epithelial cells of acute kidney injury

### 3.1 Metabolism and autophagy

Autophagy can be categorized into three types based on the process of occurrence: macroautophagy, microautophagy, and chaperone-mediated autophagy (CMA). People commonly refer to macroautophagy as autophagy. Autophagy is an evolutionarily conserved lysosomal-dependent catabolic process and serves as an important mechanism for cell homeostasis and survival under pathological stress conditions in the kidney (Decuypere et al., 2015). Many studies have shown that autophagy is triggered in renal TECs during AKI. Inhibiting autophagy, either through pharmacological means or genetic intervention, has been found to exacerbate AKI. Conversely, promoting autophagy can alleviate kidney injury (Jiang et al., 2012). However, some studies have also found that autophagy can exacerbate IR-induced kidney injury (Wu et al., 2009), indicating a dual role of autophagy in AKI. Recently, increasing evidence suggests that autophagy regulates or controls metabolism, and changes in metabolism can also affect autophagy (Yang et al., 2019).

In AKI patients, lactate generated via glycolysis downregulated the levels of phosphorylated adenosine monophosphate-activated protein kinase (p-AMPK) and SIRT3 in LPS-treated HK-2 cells. This nullified the augmented impact of 2-DG on LC2II/I in LPS-treated HK-2 cells. The hindrance of glycolysis demonstrates a capacity to enhance autophagy through the decrease of lactate production and the up-regulation of p-AMPK and SIRT3 expression. This, in turn, contributes to averting sepsis-induced AKI (Tan et al., 2021). Gatticchi et al. (2015) reported that down-regulation of FAO in PTECs during AKI can also activate autophagy, while enhancing FAO reduces the need for autophagy. Transcription factor EB (TFEB) is a key regulator of lysosomal biogenesis and autophagy and can induce lipid metabolism by regulating transcription genes involved in lipophagy and lipolysis (Nakamura et al., 2020). Gene knockout of PGC1α exacerbates the inhibitory effect of cisplatin on TFEB in mouse kidneys (Lynch et al., 2019). Mice lacking TFEB experience lipid droplet accumulation (Settembre et al., 2013). Excessive lipid levels can stimulate autophagy activity in TECs (Settembre and Ballabio, 2014). However, prolonged lipid overload can lead to lysosomal dysfunction and impairment of autophagic flux, resulting in the accumulation of phospholipids in lysosomes (Yamamoto et al., 2017). The uncoupling protein (UCP) superfamily, a critical gene for lipid degradation, is significantly downregulated in AKI mice, and levels decrease with the severity of the renal injury. Upregulation of UCP1 can alleviate lipid accumulation in AKI and significantly inhibit the progression of AKI by promoting the AMPK/unc-51 like autophagy activating kinase 1 (ULK1)/autophagy pathway (Xiong et al., 2021). Additionally, Dany and Ogretmen. (2015) have found that upregulation of the metabolite of sphingomyelin, ceramide, can induce cell death-related autophagy. On the other hand, amino acid deprivation has been shown to induce autophagy (Eleftheriadis et al., 2017). Following AKI onset, the levels of amino acids in renal tubular cells significantly decrease (Zhang et al., 2017). Up-regulation of IDO enzyme-induced tryptophan depletion can increase the autophagic flux in human renal TECs by activating eukaryotic initiation factor 2 (eIF2α) kinase general control non-derepressible 2 (GCN2) (Fougeray et al., 2012; Krupa et al., 2022). Chaudhary et al. (2015) found that increasing renal IDO1 activity or inducing autophagy with GCN2 agonists can protect mice from renal inflammatory injury. These results suggest that the up-regulation of glycolysis reduces autophagy in TECs after AKI, while the down-regulation of fatty acid oxidation, lipid accumulation, amino acid deprivation, and up-regulation of ceramide synthesis, a sphingomyelin metabolite, promote autophagy. However, abnormal accumulation of intracellular lipids can impair autophagic clearance, while the reduction of autophagic clearance further promotes lipid accumulation, exacerbating autophagy dysfunction and forming a vicious cycle. In conclusion, changes in energy metabolism of renal TECs have dual and complex effects on autophagy during AKI. Hence, it is prudent to approach the modulation of cell autophagy through the regulation of energy metabolism with caution, aiming to enhance outcomes in cases of AKI.

### 3.2 Metabolism and apoptosis

Apoptosis represents the most extensively studied form of programmed cell death in various forms of AKI. This process relies heavily on the activation of effector cysteine aspartic proteases (namely, caspase-3, caspase-6, and caspase-7) (Van Opdenbosch Lamkanfi, 2019). PK, a key enzyme in the final step of glycolysis, exists in four isoforms: PKM1, PKM2, pyruvate kinase liver isoform (PKL), and pyruvate kinase red blood cell isoform (PKR). PKM2 is significantly upregulated in the LPS-induced AKI model, and its inhibition was found to significantly suppress the expression of HIF-1α and apoptosis-related factors, such as B-cell lymphoma 2/adenovirus E1B 19 kDa interacting protein 3(BNIP3), B-cell lymphoma 2-associated X protein (Bax), and caspase-3. Moreover, it improved the pathological symptoms of LPS-induced AKI tissue, indicating that PKM2 can potentially play a role in the regulation of cell apoptosis in AKI (Wu et al., 2021). IDO upregulation leads to the depletion of tryptophan (TRP), which can activate the general control non-derepressible 2 kinases (GCN2K) pathway and inhibit the mammalian target of rapamycin (mTOR) signaling, thereby promoting renal TEC’s apoptosis. Increased IDO activity can also regulate cysteine aspartic protease-8 activation and TECs apoptosis via the Fas/Fas ligand (FasL) -dependent mechanism (Krupa et al., 2022). The metabolism of polyamines produces ROS and reactive aldehyde, which are important inducing factors for DNA damage, mitochondrial damage, endoplasmic reticulum stress/unfolded protein response, and can trigger TEC’s apoptosis and AKI renal tubular injury (Zahedi et al., 2019). Therefore, all these factors may contribute to apoptosis in AKI.

### 3.3 Metabolism and necroptosis

Necroptosis is a form of programmed cell death mediated by the phosphorylation of mixed lineage kinase domain-like protein (MLKL) by receptor-interacting protein kinase 3 (RIPK3), leading to subsequent plasma membrane rupture. In many AKI models, MLKL stands out as one of the most upregulated genes. In a clamp ischemia model, mice with defects in RIPK3 and MLKL are protected from IRI (Maremonti et al., 2022). Studies have reported elevated levels of RIPK3 in the plasma and urine of sepsis-induced AKI patients, and RIPK3 expression is also higher in AKI kidneys with tubular injury in human biopsy samples (Uni and Choi, 2022). Elevated RIPK3 can influence metabolic enzymes in the mitochondrial matrix through the RIPK1/RIPK3/MLKL necrotic body, promoting the production of mitochondrial reactive oxygen species (mtROS) that can act on glutamate dehydrogenase (GDH) and catalyze the generation of αKG from glutamate. αKG, as an energy substrate, actively participates in the respiratory chain, promotes mtROS production, and induces necroptosis (Zhao et al., 2021; Maremonti et al., 2022; Tian et al., 2022). However, Li et al. (2021) found that glycolytic metabolism inhibits RIPK-dependent necroptosis by suppressing mtROS in renal TEC injury. Therefore, further research is needed to validate the relationship between the alterations in energy metabolism of renal TECs and necroptosis.

### 3.4 Metabolism and pyroptosis

Pyroptosis is an inflammatory form of programmed cell death that primarily occurs in macrophages and dendritic cells, but it can also happen in other cell types, including hepatocytes and renal TECs. In the kidney, cellular pyroptosis can be induced by various factors, such as cadmium, contrast agents, renal ischemia-reperfusion, and unilateral ureteral obstruction, characterized by the activation of inflammatory caspase cysteine-dependent aspartate proteases and the release of interleukin-1 beta (IL-1β) (Liu et al., 2022). Studies have demonstrated that the metabolite αKG induces pyroptosis in tumor cells through death receptor6 (DR6) and gasdermin-C (GSDMC) -dependent pathways. Additionally, treatment with the end product of glycolysis, lactate, to establish acidic environments, can make cancer cells more susceptible to α-KG induced pyroptosis (Zhang et al., 2021). Hao et al. (2017) found that microRNA-17-5p (miR-17-5p) may inhibit DR-6 to promote renal TEC survival during hypoxic/ischemic renal injury, suggesting that energy metabolism in renal TECs is closely related to pyroptosis. Given the scant research in this domain, additional studies are required to substantiate these discoveries.

### 3.5 Metabolism and ferroptosis

Ferroptosis is a form of cell death regulated by iron-dependent lipid peroxidation. After hypoxia-reoxygenation, metabolites such as KYN and KYNA produced from tryptophan metabolism via the KYN pathway, along with the IDO1 enzyme from the metabolism of indoleamine, can activate aryl hydrocarbon receptor (AhR) and trigger ROS generation, leading to AhR-mediated ferroptosis of renal TECs (Krupa et al., 2022). Furthermore, during AKI, upregulation of LPCAT3 leads to phospholipid peroxidation containing polyunsaturated fatty acids, which induces ferroptosis (Zhang et al., 2022). Kang et al. (2019) found that P53-mediated ferroptosis is associated with solute carrier family 7member 11 (SLC7A11) inhibition and upregulation of SAT1 and GLS2 in cancer cells. However, further research is needed to confirm if P53 can induce iron death in AKI renal TECs by regulating these substances.

## 4 Summary and perspectives

**FIGURE 5:**
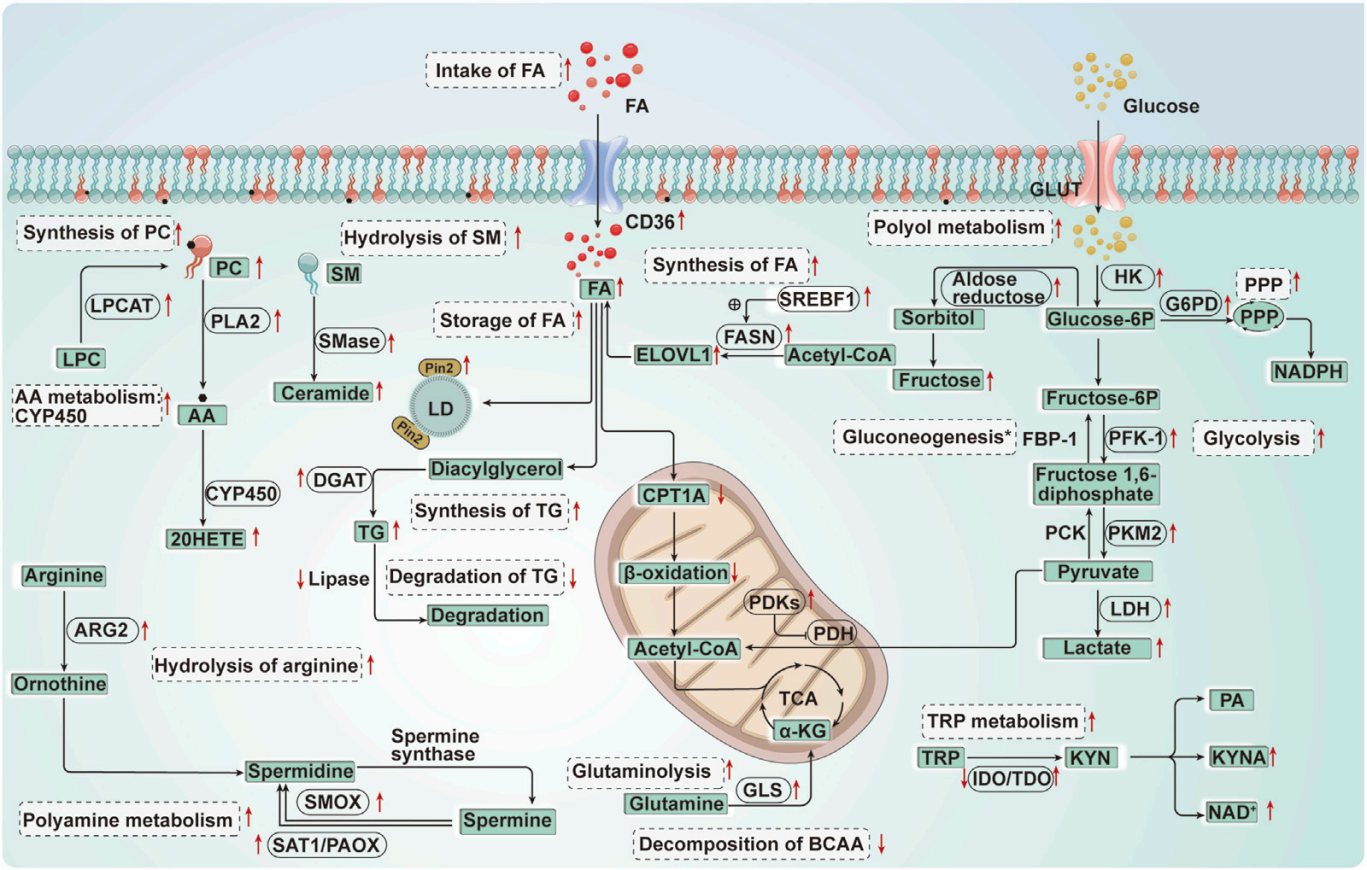
Diagram of energy metabolism changes in renal tubular epithelial cells in acute kidney injury. FA, Fatty acid; CPT1A, Carnitine palmitoyltransferase 1 A; CD36, Cluster of differentiation 36; FASN, Fatty acid synthase; SREBF1, Sterol regulatory element-binding transcription factor 1; ELOVL 1, Elongation of very long chain fatty acids like protein 1; PC, Phosphatidylcholine; LPC, Lysophosphatidylcholine; LPCAT, Lysophosphatidylcholine acyltransferase; AA, Arachidonic acid; CYP450, Cytochrome P450; 20-HETE, 20-hydroxyeicosatetraenoic acid; SM, Sphingomyelin; SMase, Sphingomyelinase; TG, Triglycerides; DGAT, Diacylglycerol acyltransferase; PLA2, Phospholipase A2; Plin2, Perilipin 2; LD, Lipid droplet; GLUT, Glucose transporters; HK, Hexokinase; FBP-1, Fructose-1, 6-bisphosphatase; PFK-1, Phosphofructokinase-1; G6PD, Glucose-6-phosphate dehydrogenase; PCK, Phosphoenolpyruvate carboxykinase; PKM2, Pyruvate Kinase M2; PDH, Pyruvate dehydrogenase; PDKs, Pyruvate dehydrogenase kinases; LDH, Lactate dehydrogenase; GLS, Glutaminase; TRP, Tryptophan; NAD, Nicotinamide adenine dinucleotide; IDO, Indoleamine 2, 3-dioxygenase; TDO, Tryptophan 2, 3-dioxygenase; KYNA, Kynurenine acid; PA, Picolinic Acid; ARG, Arginase; SAT1/PAOX, Spermidine/spermine and by N1-acetylation and N1-acetylpolyamine oxidase; SMOX, Spermine oxidase; BCAA, Branched-chain amino acid. *, Gluconeogenesis↓(FBP-1↓, PCK↓) (the acute ischemia phase of AKI); Gluconeogenesis↑ (the late reperfusion phase of AKI).↑:Up-regulation of the substance or metabolic pathway in AKI; ↓:Down-regulation of the substance or metabolic pathway in AKI. Progress of the relationship between energy metabolism reprogramming and programmed cell death in renal tubular epithelial cells of acute kidney injury.

In summary, AKI leads to significant reprogramming of energy metabolism in renal TECs. This reprogramming involves not only the well-known down-regulation of FAO and up-regulation of glycolysis but also changes in the metabolism of cell membrane lipids, triglycerides, PPP, gluconeogenesis, and amino acids (Table 1; Figure 5). FAO plays a critical role in maintaining energy supply in PTECs, occurring not only in mitochondria but also in peroxisomes. Peroxisomes not only metabolize long-chain fatty acids into shorter chain products, but also serve as a sink for the ROS generated by mitochondria due to their abundant catalase and other ROS scavenging enzymes, protecting TECs from toxic accumulation of long-chain fatty acids. Up-regulating FAO in peroxisomes has been found to protect against AKI kidney injury. Cell membrane lipid metabolism also plays an important role in AKI. LPCAT can induce ferroptosis in renal TECs by regulating phospholipid metabolism. Additionally, the metabolism of AA derivative 20-HETE and sphingolipid-derived ceramides play a significant role in AKI. Furthermore, cholesterol and triglycerides accumulate in renal TECs during AKI, inducing autophagy. Nevertheless, the abnormal accumulation of intracellular lipids hinders the autophagic clearance process. This, in turn, results in even more lipid buildup, further aggravating the impaired autophagic function. This establishes a detrimental cycle. During AKI, various changes occur in the glucose metabolism of renal TECs, including increased glycolysis, activated PPP, inhibited gluconeogenesis, and upregulated polyol metabolism. Inhibiting glycolysis and increasing PPP activity, as well as blocking the polyol pathway, may offer protective effects for the kidneys. Amino acid metabolism is also an important aspect of AKI metabolic reprogramming. Upregulated glutamine metabolism in AKI compensates for metabolic acidosis and supplements the TCA cycle. The tryptophan metabolism pathway in the kidneys mainly involves KYN, and inhibiting the PA branch of KYN metabolism while increasing the NAD branch and KYNA branch metabolism can alleviate AKI kidney injury. Moreover, the upregulation of arginase in renal TECs during AKI competes with nitric oxide synthase for the same substrate, arginine, leading to nitrosative stress and damaging cells. Branched-chain amino acid and polyamine metabolism are also closely related to AKI. These changes in energy metabolism pathways, metabolites, and key enzymes can further regulate autophagy, apoptosis, necroptosis, ferroptosis, and other pathways in renal TECs. Additionally, AKI triggers changes in nucleotide metabolism in renal TECs. Under ischemic and inflammatory conditions, ATP degrades into AMP to generate adenosine, which has been shown to have a protective effect on cells (Módis et al., 2009). Xanthine oxidoreductase (XOR) activity and purine degradation products were also found to be significantly elevated in AKI (Kosaki et al., 2022). Administering exogenous NAD and inhibiting poly ADP-ribosepolymerase (PARP) can restore renal tubular cell ATP, phosphocreatine (PCr), and NAD + levels, reducing renal TEC apoptosis (Liu et al., 2015). However, due to limited research, further experiments are needed to clarify the specific mechanisms.

**TABLE 1:** Summary of energy metabolism changes in renal tubular epithelial cells in acute kidney injury.

Energy metabolism substances and metabolic pathways			Key enzymes and products
Lipid metabolism	Fatty acid metabolism	Fatty acids oxidation↓	CPT1A↓
		The synthesis, intake, and storage of fatty acids↑	CD36↑, Perilipin 2↑, FASN↑, SREBF1↑, ELOVL1↑
	Cellular membrane lipid metabolism	Synthesis of phosphatidylcholine↑	LPCAT↑
		Hydrolysis of sphingomyelin↑	SMase↑, ceramide↑
		Arachidonic acid metabolism:CYP450↑	20-HETE↑
	Metabolism of triglycerides	Synthesis of triglycerides↑	DGAT↑
		Breakdown of triglycerides↓	lipase↓
Glucose metabolism		Glycolysis↑	HK↑, PFK↑, PKM2↑, PDKs↑, LDH↑, lactic acid↑
		The pentose phosphate pathway↑	G6PD↑
		Gluconeogenesis↓ (the acute ischemia phase of AKI)	FBP1↓, PCK1↓, PCK2↓ (the acute ischemia phase of AKI)
		Gluconeogenesis↑ (the late reperfusion phase of AKI)	
		Polyol metabolism↑	Aldose reductase↑, Fructose↑
Metabolism of amino acids	Glutamine metabolism	Glutaminolysis↑	GLS↑
	Tryptophan metabolism	Kynurenine pathway↑:the NAD branch↑, the picolinic acid branch, the KYNA branch↑	IDO↑, KMO↓, 3-HK↓
	Arginine metabolism	Hydrolysis of arginine↑	ARG2↑
	Polyamine metabolism	Polyamine degradation↑	SAT1↑, SMOX↑
	Branch chain amino acid metabolism	Decomposition metabolism of Branch chain amino acid↓	Branch chain amino acid decomposition metabolism enzymes↓

CPT1A, Carnitine palmitoyltransferase 1 A; CD36, Cluster of differentiation 36; FASN, fatty acid synthase; SREBF1, Sterol regulatory element-binding transcription factor 1; ELOVL, 1, Elongation of very long chain fatty acids like protein 1; LPCAT, lysophosphatidylcholine acyltransferase; CYP450, Cytochrome P450; SMase, Sphingomyelinase; 20-HETE, 20-hydroxyeicosatetraenoic acid; DGAT, diacylglycerol acyltransferase; PLA2, Phospholipase A2; HK, hexokinase; FBP-1, Fructose-1, 6-bisphosphatase; PFK-1, Phosphofructokinase-1; G6PD, Glucose-6-phosphate dehydrogenase; PCK, phosphoenolpyruvate carboxykinase; PKM2, Pyruvate Kinase M2; PDH, pyruvate dehydrogenase; PDKs, Pyruvate dehydrogenase kinases; LDH, lactate dehydrogenase; GLS, glutaminase; NAD, nicotinamide adenine dinucleotide; IDO, Indoleamine 2, 3-dioxygenase; TDO, Tryptophan 2, 3-dioxygenase; KYNA, kynurenine acid; KAT, kynurenine aminotransferase; KMO, Kynurenine 3-monooxygenase; 3-HK, 3-hydroxykynurenine; ARG, arginase; SAT1/PAOX, Spermidine/spermine and by N1-acetylation and N1-acetylpolyamine oxidase; SMOX, Spermine oxidase.↑, Up-regulation of the substance or metabolic pathway in AKI; ↓, Down-regulation of the substance or metabolic pathway in AKI.

Recent studies have shown that activating SIRT1 and AMPK/mToR through calorie and diet restriction enhances autophagy, counters the decrease of endothelial nitric oxide synthase (eNOS) and PGC-1α induced by I/R, and improves AKI (Lempiäinen et al., 2013; Robertson et al., 2015; Andrianova et al., 2021). Therefore, in the future, regulating the energy metabolism pathway, key enzyme activity, and metabolic product generation are potential strategies for regulating programmed cell death in renal TECs and improve AKI (Table 2). Dietary structure adjustments may also serve as supportive measures in the treatment of assist in the treatment of AKI.

**TABLE 2:** Summary of the literature on the regulation of energy metabolic reprogramming to improve AKI.

Energy metabolism substances	Targeted energy metabolic pathways	Targets	Interventions	Injury model	References
Lipid metabolism	Fatty acids oxidation↑	CPT1A↑	C75	I/R	Idrovo et al. (2012)
			PPARγ agonist	I/R	Hu et al. (2012)
			PGC1α agonist	I/R	Wang et al. (2021)
			PXR agonist	I/R	Yu et al. (2020)
	Synthesis of S1P↑	S1P↑, SK-1↑	Selective A1AR agonist (CCPA)	I/R	Park et al. (2012)
	Synthesis of ceramides↓	Sphingomyelinase↓	Myriocin, amitriptyline	Cisplatin	Dupre et al. (2017)
	Arachidonic acid metabolism:CYP450	20-HETE↑	20-HETE agonists	I/R	Regner et al. (2009)
		20-HETE↓	20-HETE Inhibitors	I/R	Hoff et al. (2011)
Glucose metabolism	Glycolysis↓, pentose phosphate pathway↑	PKM2↓	Shikonin, S-nitroso-CoA	Lipopolysaccharide, I/R	Wu et al. (2021); Zhou et al. (2019)
	Gluconeogenesis↓	Reduction in insulin resistance	Insulin	I/R	Melo et al. (2010)
			Glipizidone	I/R	Hu et al. (2012)
	Polyol metabolism↓	Fructokinase↓	luteolin	I/R	Andres-Hernando et al. (2017)
Amino acid metabolism	Tryptophan metabolism: Kynurenine pathway↑:the NAD branch↑, the picolinic acid branch↓, the KYNA branch↑	ACMSD↓	ACMSD inhibitors	Cisplatin	Katsyuba et al. (2018)
		KYNA↑	Exogenous supplementation with KYNA	I/R	Arora et al. (2014)
		KMO↓	KMO gene blockade	I/R	Zheng et al. (2019)
		NAD↑	PGC1α agonist	I/R	Tran et al. (2016)
	Arginine metabolism: arginase pathway↓	Arginase2↓	Arginase2 inhibitors	I/R	Hara et al. (2020)
	Arginine metabolism: nitric oxide synthase pathway↑	Arginase2	Exogenous administration of arginine	Sepsis	Tanuseputeroet al. (2020)
	Polyamine degradation↓	SAT↓, SMOX↓	Ablation of the SSAT or SMOX gene	Cisplatin, Sepsis	Zahedi et al. (2010); Zahedi et al. (2017)

FAO: CPT1A, Carnitine palmitoyltransferase 1 A; PGC1α, Peroxisome proliferator-activated receptor coactivator (PGC)-1 alpha; PPARγ, Peroxisome proliferator-activated receptor-gamma; PXR, pregnane X receptor; CYP450, Cytochrome P450; 20-HETE, 20-hydroxyeicosatetraenoic acid; PKM2, Pyruvate Kinase M2; SK-1, Sphingosine kinase 1; S1P, Sphingosine-1-phosphate; NAD, nicotinamide adenine dinucleotide; KYNA, kynurenine acid; KMO, Kynurenine 3-monooxygenase; SAT1/PAOX, Spermidine/spermine and by N1-acetylation and N1-acetylpolyamine oxidase; SMOX, spermine oxidase; ACMSD, aminocarboxymuconate semialdehyde decarboxylase; I/R:Ischemia/reperfusion. ↑, Up-regulation of the substance or metabolic pathway after the intervention; ↓, Down-regulation of the substance or metabolic pathway after the intervention.

## Figures and Tables

**Table:** 

**AKI**	Acute kidney injury; TECs, Tubular epithelial cells; PTECs, Proximal renal tubular epithelial cells
**FAO**	Fatty acid oxidation
**PPP**	Pentose phosphate pathway
**IRI**	Ischemia-reperfusion injury
**ROS**	Reactive oxygen species
**FAs**	Fatty acids
**CPT**	Carnitine palmitoyltransferase
**Acyl-CoA**	Acyl-Coenzyme A
**TCA**	Tricarboxylic acid
**NADH**	Nicotinamide adenine dinucleotide hydrogen
**FADH**	Flavin Adenine Dinucleotide hydrogen
**Sirt**	Sirtuin
**PGC**	Peroxisome proliferator-activated receptor coactivator
**PGC1α**	Peroxisome proliferator-activated receptor coactivator -1 alpha
**PPAR**	Peroxisome proliferator-activated receptor
**CD36**	Cluster of Differentiation 36
**LPCAT**	Lysophosphatidylcholine acyltransferase
**20-HETE**	20-hydroxyeicosatetraenoic acid
**19-HETE**	19-hydroxyeicosatetraenoic acid
**I/R**	Ischemia/reperfusion
**FFAs**	Free fatty acids
**LPS**	Lipopolysaccharide
**LDH**	Lactate dehydrogenase
**HK**	Hexokinase
**PFK**	Phosphofructokinase
**PK**	Pyruvate kinase
**PDH**	Pyruvate dehydrogenase
**HIF-1α**	Hypoxia-inducible factor-1α
**PKM**	Pyruvate kinase muscle isoform
**NADPH**	Nicotinamide adenine dinucleotide phosphate
**GSH**	Glutathione
**G6PD**	Glucose-6-phosphate dehydrogenase
**αKG**	α-ketoglutarate
**NOS**	Nitric oxide synthase
**KYN**	Kynurenine
**NAD**	Nicotinamide adenine dinucleotide
**GLS**	Glutaminase
**KP**	Kynurenine pathway
**IDO**	Indoleamine 2,3-dioxygenase
**TDO**	Tryptophan 2,3-dioxygenase
**KYNA**	Kynurenic acid
**SAT1/PAOX**	Spermidine/spermine and by N1-acetylation and N1-acetylpolyamine oxidase
